# 

**Published:** 1803-10-01

**Authors:** 


### TO CORRESPONDENTS.

Tynicola's reply to Dr. Mossman, and Empiricus's to Dr. Magennis on Apoplexy, are postponed for want of proper signatures.

Communications are received from Mr. Weber, Mr. Thoresby, and Mr. Marson.

Mr. Smith's request is complied with.

